# Supplementary material for: Wound complications following surgery to the lymph nodes: A protocol for a systematic review and meta-analysis
Source: PLoS One. 2022 Aug 3;17(8):e0272490. doi: 10.1371/journal.pone.0272490 (PMC9348652; doi:10.1371/journal.pone.0272490)
Supplement: Attachment — Submitted filename: Reply to Reviewers.docx [file pone.0272490.s003.docx]

13^9h^ July 2021

Dear Editors,

Thank you for your review of our manuscript, *Wound Complications Following Surgery to the Lymph Nodes: A protocol for a systematic review and meta-analysis.*

We were confused by the content of the review. In the review, the article is deemed to meet the criteria for publication (The reviewer’s responses to all questions is “Yes”). It clearly states that the manuscript is a protocol, and was submitted as such during the submission process. However, the reviewer has refused to accept the article, citing “Evaluating an article without the Results section is quite confusing. It would be appropriate to re-evaluate after the process and the article are completed.”

We feel the reviewer has not understood that the article is a review protocol, rather mistaking it for a half completed systematic review. As such, we have no substantial changes to make based on the reviewers comments.

Please find our responses to each individual point:

***Editors Comments:***

1. Please ensure that your manuscript meets PLOS ONE's style requirements, including those for file naming.

*Amended and updated. Thanks.*

2. We note that you have stated that you will provide repository information for your data at acceptance. Should your manuscript be accepted for publication, we will hold it until you provide the relevant accession numbers or DOIs necessary to access your data. If you wish to make changes to your Data Availability statement, please describe these changes in your cover letter and we will update your Data Availability statement to reflect the information you provide.

*Data availability applies to the completed review. As this submission is a study protocol, no data will be generated and therefore the submission should not be withheld. Full data in a repository will be provided with the completed review in a separate submission. Thanks*

3. Please include captions for your Supporting Information files at the end of your manuscript, and update any in-text citations to match accordingly. Please see our Supporting Information guidelines for more information: http://journals.plos.org/plosone/s/supporting-information.

*Amended and Updated. Thanks.*

***Reviewer’s Comments:***

Thank you for the opportunity to review this article. It is a well planned and structured meta-analysis. However authors declerated that results will be undertaken after the registration of the protocol on PROSPERO. Evaluating an article without the Results section is quite confusing. It would be appropriate to re-evaluate after the process and the article are completed.

*Thank you for your comments. The protocol is registered on PROSPERO and the registration information is contained within the manuscript. This is a study protocol and as such no results have been generated. Publishing protocols is deemed good practice and is practiced by the Cochrane Collaboration. Results will be available in due course once the study has been completed, and will be submitted to an appropriate peer reviewed journal.*

As per your email of 14^th^ July, please amend the funding statement to “The authors received no specific funding for this work.”

We have resubmitted the article with some formatting changes as requested by the journal. We fail to understand, based on this review, why our article would be rejected for PLOS ONE: Protocols, when there was no significant criticism of the protocol we presented. We hope to receive your response in a timely fashion.

Kind regards,

Joshua Totty

MBBS, PGCertRes, MRCS, MD, FHEA

Registrar in Plastic and Reconstructive Surgery (ST3) - Hull University Teaching Hospitals NHS Trust

Yorkshire and Humber Deanery

NIHR Clinical Lecturer in Plastic Surgery

Hull York Medical School
