# Supplementary material for: Wound complications following surgery to the lymph nodes: A protocol for a systematic review and meta-analysis
Source: PLoS One. 2022 Aug 3;17(8):e0272490. doi: 10.1371/journal.pone.0272490 (PMC9348652; doi:10.1371/journal.pone.0272490)
Supplement: Attachment — Submitted filename: Reply to reviewers.docx [file pone.0272490.s005.docx]

Reply to reviewers:

1. Please note that in order to use the direct billing option the corresponding author must be affiliated with the chosen institute. Please either amend your manuscript to change the affiliation or corresponding author, or email us at plosone@plos.org with a request to remove this option.

Thank you. I can confirm that as the corresponding author I am affiliated with the University of Hull, listed in my affiliations in the manuscript.

2. Thank you for stating the following in the Funding Section of your manuscript:

“No direct funding was secured for this protocol or subsequent review. Joshua Totty is a Clinical Lecturer funded by Health Education England (HEE) / National Institute for Health Research (NIHR). The views expressed in this publication are those of the author(s) and not necessarily those of the NIHR, NHS or the UK Department of Health and Social Care.”

We note that you have provided funding information within the funding Section that is not currently declared in your Funding Statement. Please note that funding information should not appear in the Acknowledgments section or other areas of your manuscript. We will only publish funding information present in the Funding Statement section of the online submission form.

Please remove any funding-related text from the manuscript and let us know how you would like to update your Funding Statement. Currently, your Funding Statement reads as follows:

“The authors received no specific funding for this work.”

Please include your amended statements within your cover letter; we will change the online submission form on your behalf.

Thank you. The funding statement should read “No direct funding was secured for this protocol or subsequent review. Joshua Totty is a Clinical Lecturer funded by Health Education England (HEE) / National Institute for Health Research (NIHR). The views expressed in this publication are those of the author(s) and not necessarily those of the NIHR, NHS or the UK Department of Health and Social Care”

3. Please review your reference list to ensure that it is complete and correct. If you have cited papers that have been retracted, please include the rationale for doing so in the manuscript text, or remove these references and replace them with relevant current references. Any changes to the reference list should be mentioned in the rebuttal letter that accompanies your revised manuscript. If you need to cite a retracted article, indicate the article’s retracted status in the References list and also include a citation and full reference for the retraction notice.

Thank you. I can confirm the reference list is up to date.

Reviewer #2: The protocol is well presented with appropriate details. Authors have mentioned the aim of the study. It would be more clearer if there is any mention on the study objectives and what are the specific review questions. These could be spelt clearly before describing the methods. Systematic reviews and protocols generally have the eligibility criteria mentioned in the PICO framework (also mentioned in the PRISMA-P). The authors need to mention this in the protocol.

We thank the reviewer for their comments. We have updated and amended the manuscript as suggested, with the addition of a section titled “study objectives”, including a PICO framework, before the methods section.
